# Reinventing the antimicrobial pipeline in response to the global crisis of antimicrobial-resistant infections

**DOI:** 10.12688/f1000research.18302.1

**Published:** 2019-03-01

**Authors:** Andrew C. Singer, Claas Kirchhelle, Adam P. Roberts

**Affiliations:** 1 NERC Centre for Ecology & Hydrology, Wallingford, OX10 8BB, UK; 2 Wellcome Unit for the History of Medicine, University of Oxford, Oxford, OX2 6PE, UK; 3 Antimicrobial Chemotherapy and Resistance, Liverpool School of Tropical Medicine, Liverpool, L3 5QA, UK

**Keywords:** antibiotics, antibiotic resistance, antimicrobial, drug pipeline

## Abstract

The pipeline for new antibiotics is dry. Despite the creation of public/private initiatives like Combating Antibiotic Resistant Bacteria Biopharmaceutical Accelerator (Carb-X) and the Antimicrobial Resistance (AMR) Centre, the current focus on ‘push-pull’ incentives for the pharmaceutical industry still relies on economic return. We propose a joint, internationally-funded antimicrobial development institute that would fund permanent staff to take on roles previously assigned to pharmaceutical companies. This institute would receive ring-fenced, long-term, core funding from participating countries as well as charities, with the aim to focus on transforming the largely dormant antimicrobial pipeline. Resulting drugs would be sold globally and according to a principle of shared burdens. Our proposed model for antimicrobial development aims to maximise society’s investment, through open science, investment in people, and the sharing of intellectual property.

The UK’s new five-year national antimicrobial resistance (AMR) action plan highlights that society is at a tipping point: not only are we running out of effective antibiotics, but the pipeline for new drugs is dry and novel diagnostics are slow to come to market ( HM Government, 2019). Over the past two decades, decision-makers have tried to overcome this dry spell by relying on the market and creating ‘push-pull’ incentives for the pharmaceutical industry ( Renwick *et al*., 2016). The fundamental premise of ‘push-pull’ incentives, is to make it economically attractive for major pharmaceutical companies to invest their infrastructure, staff and skills into the research, development and manufacture of novel antibiotics. The reality for pharmaceutical companies is that there is too little money to be made with antibiotics: any new antibiotic will be reserved as a ‘last-line defence’, which means it will (hopefully) be an infrequently used antibiotic and as such, a poor source of income.

While recent public-private initiatives like Combating Antibiotic Resistant Bacteria Biopharmaceutical Accelerator (Carb-X) and the AMR Centre (UK), are important steps in the right direction, we strongly contend that using public funds to support private venture and profit is not the only way to refill the antibiotic pipeline. In the current model for drug development, the tax payer pays for everything up to the point where the pharmaceutical companies may invest in later stages of development ( Galkina Cleary *et al*., 2018). Society invests in the students, post-docs, and professors through public funding of the university and/or the research grant that pays their salaries and equipment. However, at the point where knowledge becomes patentable, it quickly disappears down pharmaceutical pipelines.

Commercial pipelines are not always efficient producers of new drugs. Potentially promising drugs are abandoned when commercial or global health priorities change. In some cases, patents are only filed to deter competitors from developing them further. Not only are society’s investments in antimicrobial innovation wasted, but the intellectual property associated with a drug’s development, i.e., the countless serendipitous leads as well as dropped, yet promising, antimicrobials, remain locked-up within the private pharmaceutical company—inaccessible to the public who invested in the initial development. Ultimately, the current mode of subsidised antibiotic development means that society pays for 100% of all the drugs that are developed and not developed, but, importantly, owns and controls nothing (discussed here ( Anon, 2019)). It is this obvious disconnect in societal investment, which needs to be reformed.

We propose a radical change to the current paradigm of antimicrobial development and manufacture—one that reflects humanity’s shared interests and global health challenges. We propose a joint, internationally-funded antimicrobial development Institute that would fund permanent staff to take on the role previously assigned to pharmaceutical companies. This institute would receive ring-fenced, long-term, core funding from participating countries as well as charities, with the aim to focus on transforming the largely dormant antimicrobial pipeline.

The international centre would aim to sustainably develop a breadth of new antimicrobials to address both immediate and emerging global health challenges, respond to needs across organisms, i.e., viral, bacteria, fungal, protist, and develop novel modes of action. Prospective antimicrobials submitted to the centre would be developed in an open and transparent manner, so that innovation can be immediately shared and serendipitous findings can be leveraged by the wider research community. The centre would also conduct clinical trials on prospective drug candidates, manufacture all antimicrobials through existing generic drug manufacturers, and contract research organisations.

Resulting drugs would be sold globally and according to a principle of shared burdens. This would mean that high-income countries would pay more for research and the drugs themselves than low-income countries facing ongoing access problems. Signing up to enforceable stewardship requirements would be a precondition to receiving new drugs. Since no development costs need recouping and no share holder incentives need to be satisfied, most drugs could be sold at the cost of manufacture. Nearly 40 years of lacking commercial interest in new antibiotic development means that our ‘not-for-profit’ antibiotic pipeline would not compete with established manufacturers.

A look back at the 20 ^th^ century shows that our proposed approach is not as far-fetched as it may sound. While a turn to more profitable ‘lifestyle’ drugs ended pharmaceutical investment in antibiotic development from the late 1970s onwards ( Gradmann, 2016), the 1940s saw many of the same companies work hand-in-hand with nation states and universities to screen antimicrobial compounds, test them in clinical settings, and upscale production. There was no patent on penicillin – only on the process developed to mass-produce it ( Bud, 2009). In the case of antimalarials, military interests led to a long history of state-directed and subsidized development ( Lezaun, 2018). 

Our proposed model for antimicrobial development aims to maximise society’s investment, through open science, investment in people, and the sharing of intellectual property. Antimicrobial resistant infections pose a global challenge. It is time to realise that the challenge of solving the global problem of AMR exceeds the capacity of commercial actors. We are already financing the development of antibiotics, why not collectively own and manage the resulting drugs?

## Data availability

No data are associated with this article
