# Supplementary material for: Reinventing the antimicrobial pipeline in response to the global crisis of antimicrobial-resistant infections
Source: F1000Res. 2019 Mar 1;8:238. [Version 1] doi: 10.12688/f1000research.18302.1 (PMC6426076; doi:10.12688/f1000research.18302.1)
Supplement: Peer review history

# Peer review: Reviewer response for version 1

**DOI:** 10.5256/f1000research.20020.r45713

**Published:** 2019-03-19

**Authors:** Dean Baker

**Affiliations:** 1 Center for Economic and Policy Research (CEPR), Washington, DC, USA

This article proposes to establish an international center for antimicrobial research and development. The center would develop and then sell drugs to according to a principle of "shared burden." There is a need for such a center, since the private pharmaceutical industry has largely abandoned research in this area since it sees little prospect for profit. 

 While this is an innovate idea, it would be helpful to see it flushed out a bit more in two directions. First, while it seems the authors' intentions that all research findings be open, a slightly clearer statement on this principle would be helpful. For example, do they envision something like the Bermuda Principle, where results are posted to the web, if not nightly, as quickly as practical.

 The second area that could benefit from clarification is the meaning of the principle of "shared burden." Since the research costs are being paid upfront, it is unclear why there would be any reason to charge a price above the marginal cost. (There could an issue where countries that did not contribute to research costs get charged more, but that should be explicitly stated.)

 Anyhow, this is a worthwhile proposal which should advance the debate on the best way to finance the development of prescription drugs.

I have read this submission. I believe that I have an appropriate level of expertise to confirm that it is of an acceptable scientific standard.

# Peer review: Reviewer response for version 1

**DOI:** 10.5256/f1000research.20020.r45186

**Published:** 2019-03-13

**Authors:** Christoph Gradmann

**Affiliations:** 1 Section for Medical Anthropology and Medical History, Institute of Health and Society, University of Oslo, Oslo, Norway; 2 Centre de recherche, médecine, sciences, santé, santé mentale, société (Cermes3), EHESS - Université Paris Descartes, Villejuif, France

The authors have written a bold, interesting and well argued opinion piece. It discusses alternatives to reviving antibiotics drug development beyond the ‘push-pull’ incentives offered to pharmaceutical industry. They would essentially result in making novel antibiotics a public commodity. This should trigger much needed debate.

 I have two comments. One is in detail. In the opening of the third last paragraph we find the sentence "Resulting drugs would be sold globally ...". Is that consistent with the approach. After all selling could again make the invented drugs private property and prohibit keeping their further development in open areas. Would not licensing be better?

 My second question is about comparable stories. I am no great expert in vaccines but it seems to me that in this case we have seen rather successful cases of public-private partnerships in the last decades. Maybe worth considering. Historical reading could start from "Galambos, Louis, and Jane Eliot Sewell. Networks of Innovation: Vaccine Development at Merck, Sharp and Dohme, and Mulford, 1895-1995. Cambridge: Cambridge University Press, 1996."

I have read this submission. I believe that I have an appropriate level of expertise to confirm that it is of an acceptable scientific standard.
